# New Challenges in Assessment of the Acoustic Properties of Coating Polymers

**DOI:** 10.3390/polym17101418

**Published:** 2025-05-21

**Authors:** Mariana Domnica Stanciu, Maria Violeta Guiman, Silviu Marian Năstac

**Affiliations:** 1 Department of Mechanical Engineering, Transilvania University of Brașov, B-dul Eroilor 29, 500036 Brasov, Romania; violeta.guiman@unitbv.ro (M.V.G.); silviu.nastac@ugal.ro (S.M.N.); 2 Faculty of Engineering and Agronomy, Braila, “Dunarea de Jos” University of Galati, 999074 Braila, Romania

**Keywords:** coating polymers, acoustic properties, resonance spruce, modal analysis, impedance tube

## Abstract

The study presented in this paper investigates the influence of coating polymers on the acoustic properties of resonant spruce wood. It evaluates absorption, acoustic reflection, and resonance frequency spectrum characteristics in both unvarnished and varnished samples, with the interface between the coating polymer and the wood modifying the acoustic response. The novelty of the research consists in evaluating the acoustic and dynamic parameters of resonant spruce wood boards, varnished with varnishes with different chemical properties (oil-based varnish, spirit varnish, nitrocellulose varnish). The study focuses on the influence of the type of varnish and the thickness of the varnish film on the frequency spectrum, damping coefficient, quality factor, acoustic absorption coefficient, and sound reflection. The sound absorption coefficient increases with the number of varnish layers and is influenced by the sound’s frequency range, the type of varnish, and the quality of the wood—factors that collectively enhance acoustic performance. For instance, oil-based varnish applied in 5 or 10 layers contributes to a fuller sound at a frequency of 1.5 kHz. In contrast, spirit varnish, which has a lower acoustic absorption coefficient at this frequency, and a reduced damping coefficient, can lead to a nasal tone, although the frequency spectrum turns out to have the richest. Applying more than 10 layers of varnish softens the sound when using oil-based varnish but sharpens it with spirit varnish on resonant wood. Thus, the acoustic performance of a soundboard can be tailored by selecting the appropriate varnishing system and number of layers applied. However, a detailed analysis of the timbre of musical instruments finished with these varnishes is necessary to confirm their influence on the acoustic quality of the instruments.

## 1. Introduction

**Figure 1:**
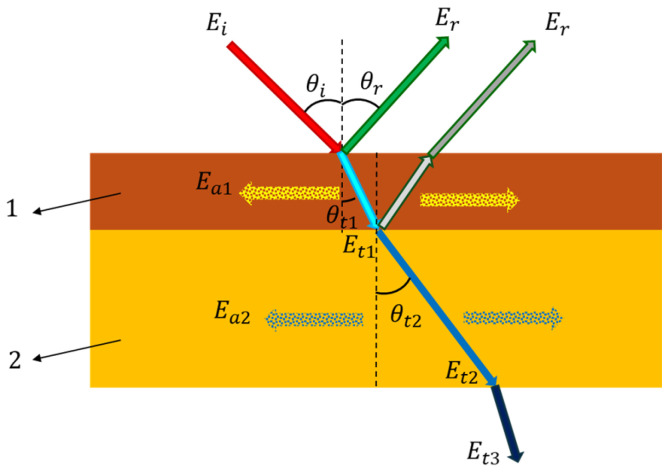
Sound wave incident into a material with layers having different elastic and absorbing properties.

Most studies on resonant spruce have focused on analyzing its acoustic properties from the perspective of sound propagation speeds in the three principal directions, the quality factor, damping coefficient, resonance frequencies, and acoustic radiation [1,2]. Softwoods used in violin top plates exhibit a high level of acoustic performance, characterized by high sound propagation speeds in the longitudinal direction (around 5500 m/s), high elastic moduli (aprox. 14–15 GPa), strong acoustic radiation, and efficient acoustic energy conversion [1,2]. Resonance spruce wood also displays moderate acoustic impedance, moderate to low density, low damping, and high anisotropy—meaning a significant variation in properties depending on the direction [3,4,5,6]. However, the acoustic and vibrational phenomena in violin wood are complex, as sound energy is transformed into absorbed, reflected, and transmitted energy [7,8,9,10]. Consequently, the anatomical structure of the wood has a crucial role in the acoustics of the musical instrument, as demonstrated in numerous studies [11,12,13,14]. In addition, the wood–varnish system significantly influences the transmission of acoustic energy. This system forms two distinct environments through which sound travels, with the type of varnish being a defining factor. From a material structure perspective, the timbre of a violin results from a layered system composed of the wood (substrate), the wood–varnish interface, and the varnish film (coating) [15,16,17,18]. While the interior of the violin body remains unvarnished, the exterior is fully coated with varnish, leading to different absorption and reflection phenomena at the interfaces between varnished wood and external air, and between unvarnished wood and the air inside the resonator. Studies [19,20,21,22,23,24] have shown that sound energy absorption in wood is accompanied by mechanical, acoustic, and thermal phenomena. Structurally, the number of pores (cavities, channels, and interstices), their dimensions, interconnections, and the interface between external surfaces and internal pores are key characteristics that affect the absorption, reflection, and transmission of acoustic energy. These aspects have been the focus of numerous studies [25,26,27,28]. Yin and Guan (2024) [29] emphasized the influence of structural parameters on sound absorption performance, depending on the positioning of layers with varying elastic and porous properties relative to the incident acoustic energy. Figure 1 illustrates how incident acoustic energy (E_i_) is reflected (E_r_), absorbed (E_a1_, E_a2_), and transmitted (E_t1_, E_t2_, E_t3_) through the media it encounters—namely the lacquer film (1) and the wood (2), which together form a layered system with distinct material properties.

The relationship between the acoustic properties of resonant wood and the different types of varnishes applied has been analyzed from physical, elastic, morphological, and chemical perspectives, as highlighted in studies [28,29,30]. However, it has been found that the existing literature is limited in terms of research on the effects of coatings—particularly the type and thickness—on the sound absorption coefficients of solid wood materials such as violin soundboards.

Previous studies [30,31] focused on characterizing the morphology of both unvarnished and varnished surfaces of resonant wood, as well as determining the acoustic absorption and reflection coefficients for materials coated with oil-based and alcohol-based varnishes. The acoustic absorption coefficient indicates the material’s ability to absorb sound waves across various frequencies. In contrast, the reflection of sound waves refers to the phenomenon in which waves return to the medium from which they originated upon encountering a boundary with another medium of different density. To characterize this reflection phenomenon, a quantity known as the reflection coefficient—or acoustic reflection factor—is introduced and determined empirically. The reflection coefficient is defined as the ratio between the amplitude of the reflected wave and the amplitude of the incident wave.

The novelty of the research presented in this article lies in the comparative analysis of acoustic parameters—specifically sound absorption and reflection coefficients—as influenced by varnish film thickness, varnish type (oil-based varnish, spirit varnish, nitrocellulose varnish), and the anatomical quality of spruce wood. The acoustic absorption and reflection coefficients were determined using the transfer-function method.

## 2. Materials and Methods

### 2.1. Samples Preparation

**Figure 2:**
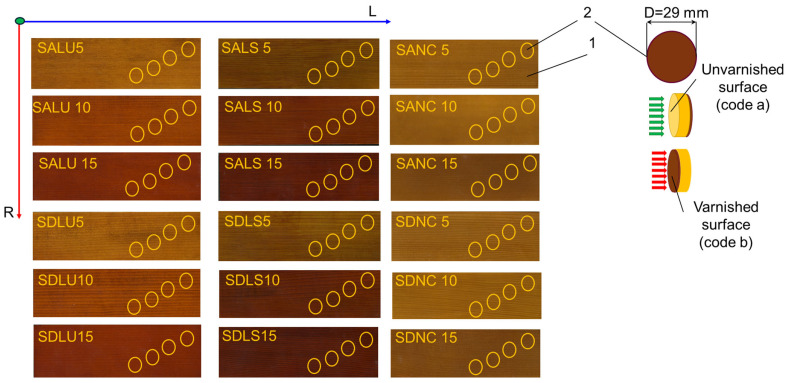
The specimens prepared for study: (Legend: 1—sample for modal analysis test; 2—sample for impedance tube test; D—sample diameter; L—longitudinal direction of wood; R—radial direction of wood).

In the first stage, 18 spruce wood boards with anatomical and physical characteristics typical of resonant wood—classified as quality grades A and D—were prepared, as shown in Table 1 [31]. The unvarnished spruce plates from grades A and D (denoted SA and SD), with a moisture content of 6–8% and dimensions of 240 × 80 × 4 mm^3^, were varnished using three types of varnish: oil-based varnish (coded LU), spirit varnish (LS), and nitrocellulose varnish (NC). Each varnish type was applied in three different layer thicknesses related to the number of layers: 40 ± 10 μm (coded 5), 80 ± 10 μm (coded 10), and 130 ± 10 μm (coded 15). The base coat was sanded using 280-grit sandpaper, and each subsequent varnish layer was sanded with 320-grit sandpaper. The varnish application was carried out at a musical instrument factory, following standard lacquering procedures used for violins. From each type of board, four circular samples with a diameter of 29 mm were extracted for testing using the impedance tube method, as illustrated in Figure 2. Table 2 presents the types of samples prepared for the acoustic impedance tube and their physical characteristics.

### 2.2. Experimental Set-Up

#### 2.2.1. The Modal Analysis of Varnished Plates

**Figure 3:**
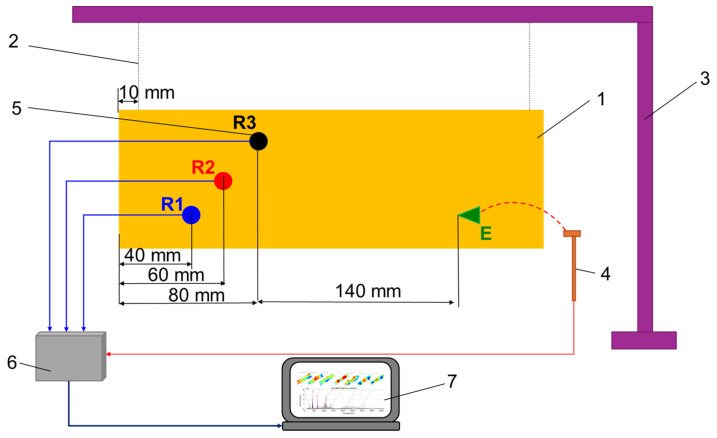
Experimental set-up for modal analysis (Legend: 1—sample; 2—elastic supports; 3—rigid frame of experimental bench; 4—impact hammer (applied in point, denoted E); 5—accelerometers (denoted with signal R1, signal R2, signal R3); 6—dynamic data acquisition board; 7—laptop).

Experimental modal analysis of spruce wooden plates (1) before varnish (coded SA and SD) and after finishing (coded SALU, SDLU, SALS, SDLS, SANC, SDNC) consisted of exciting them with an impact hammer type B&K 8204 (4) [26,27,31,32,33]. Each plate was supported by elastic elements (2), thus simulating a system with a free structure (Figure 3). For the elastic supports (2) of the plates, a rigid frame was used, with a cantilever beam (3).

**Figure 4:**
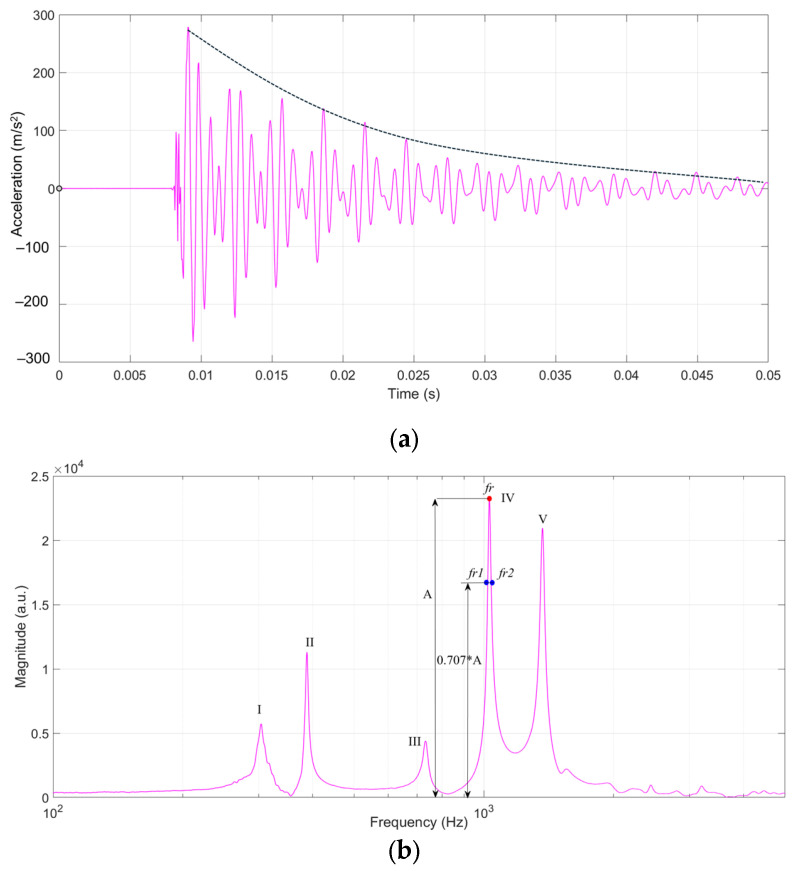
Schematic of the method of extracting quantities for calculating the logarithmic decrement and the quality factor: (**a**) time domain analysis; (**b**) frequency spectrum (I, II, III, IV, V—resonance frequency (*f_r_*), frequencies $f_{r1}$ $f_{r2}$ extracted at 0.707 from the magnitude).

The resulting signal was received by means of three accelerometers of the type B&K 4517–002 (5), placed according to the scheme shown in Figure 3. The generated signals from R1, R2, and R3 accelerometers were passed through a signal conditioner to a NI USB-9233 dynamic data acquisition board (6) manufactured by National Instruments (Austin, TX, USA), and connected to a laptop (7). The signal was processed using an application developed in MatLab© software, R2024a version (Figure 3). To obtain the values of the fundamental frequencies and the damping factor, all the signals from R1, R2, and R3 accelerometers were graphically processed, resulting in an analysis in time and frequency. The tests were carried out in the laboratory, under controlled conditions of temperature (22+/−2 °C) and relative air humidity (55+/−5%). From frequency analysis, the logarithmic decrement tanδ (dimensionless) was determined, which represents the internal friction manifested by the loss of mechanical energy in the process of vibration propagation, knowing the resonance frequency $f_{r}$ and its amplitude as can be seen in Figure 4. Thus, for each resonance frequency in the frequency spectrum, the two frequencies $f_{r1}$ $f_{r2}$ were determined, extracted at 0.707 from the magnitude of each resonance frequency, as seen in Figure 4, after which the values were introduced into the logarithmic decrement relation (1) [33,34]. The quality factor Q was calculated with the relation (2) [33,34].

(1) $$tan\delta =\frac{\left(f_{r2}-f_{r1}\right)}{f_{r}},$$

(2) $$Q=\frac{1}{\tan\delta }$$

#### 2.2.2. The Impedance Tube Method

**Figure 5:**
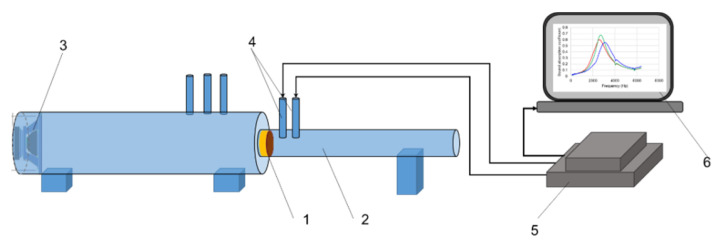
Experimental set-up for acoustic impedance tube method (1—sample; 2—impedance tube; 3—speaker; 4—microphone; 5—data acquisition board; 6—computer).

The sound absorption coefficient (denoted SAC) of the samples was measured using an impedance tube, following the standard procedures outlined in standards ISO 11654:1997, ISO 10534-2/1998 and ASTM E1050-12/1998. These methods are based on the transfer function method with two microphones (TFM) [34,35,36,37]. The impedance tube enables measurements under normal incidence conditions (zero-degree angle) with a controlled frequency band for the incident acoustic waves. The sound reflection coefficient (SRC) was also evaluated. This parameter depends on the material’s surface profile, the frequency of the traveling wave, and the angle of incidence—that is, the angle between the wavefront and the normal surface [35,36,37,38,39]. The general setup used to evaluate the sound absorption and reflection coefficients is illustrated schematically in Figure 5. Each circular sample, with a diameter of 29 mm (1), was placed into the sample holder of an impedance tube (2), model 4206 from Brüel & Kjær (Nærum, Denmark). Acoustic signals in the frequency range of 100 Hz to 6.4 kHz were generated inside the tube using a speaker (3). The signals captured by the microphones (4) were processed via a signal conditioner and DAQ data acquisition board (5),and subsequently analyzed using a computer system (6). Each sample was tested on both its unvarnished and varnished surfaces to identify differences in sound absorption coefficients attributable to the varnish layers [34]. Accordingly, when coding the samples, the notation “a” was used for the unvarnished surface exposed to sound pressure from the impedance tube, and “b” for the varnished surface. The evaluation was based on the measured minimum and maximum sound pressure levels. In total, 144 measurements were performed. All tests were conducted in a laboratory environment with controlled conditions: temperature (22 ± 2 °C) and relative humidity (55 ± 5%).

#### 2.2.3. The Processing Data

Based on experimental data, the resonance frequencies, damping, quality factor, reflection coefficient, and impedance ratio were analyzed in relation to the thickness and type of varnish. The data processing involved calculating quantitative variables, including average, minimum, and maximum values, as well as the coefficient of variation.

## 3. Results and Discussion

### 3.1. Resonance Frequencies Spectrum

**Figure 6:**
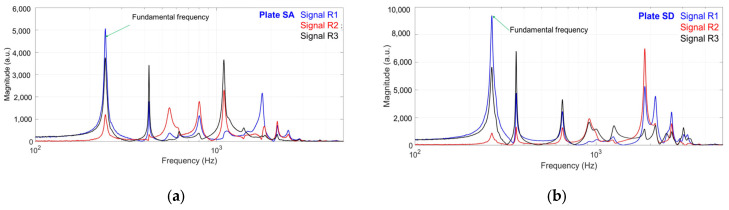

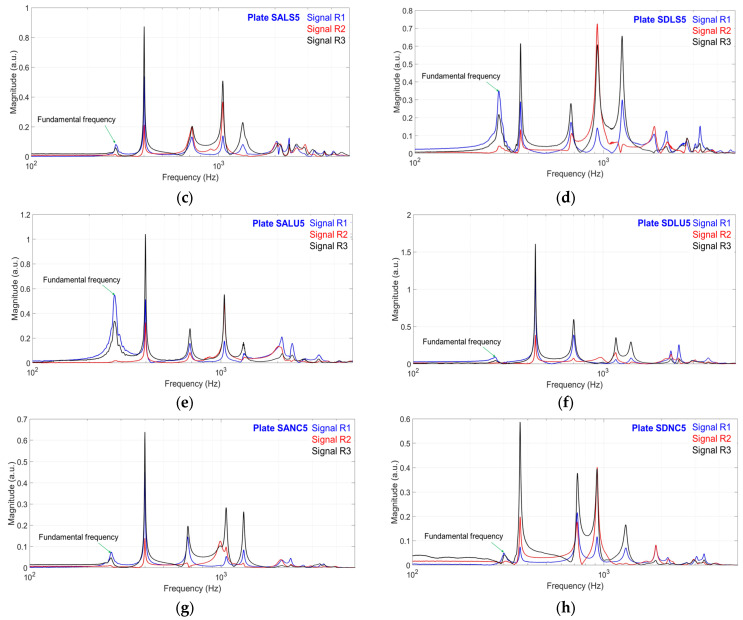
Frequency spectrum of tested plates: (**a**) unvarnished plate, grade A; (**b**) unvarnished plate grade D; (**c**) spirit varnish plate, grade A; (**d**) spirit varnish plate, grade D; (**e**) oil-based varnish plate, grade A; (**f**) oil-based varnish plate, grade D; (**g**) nitrocellulose varnish plate, grade A; (**h**) nitrocellulose varnish plate, grade D.

**Figure 7:**
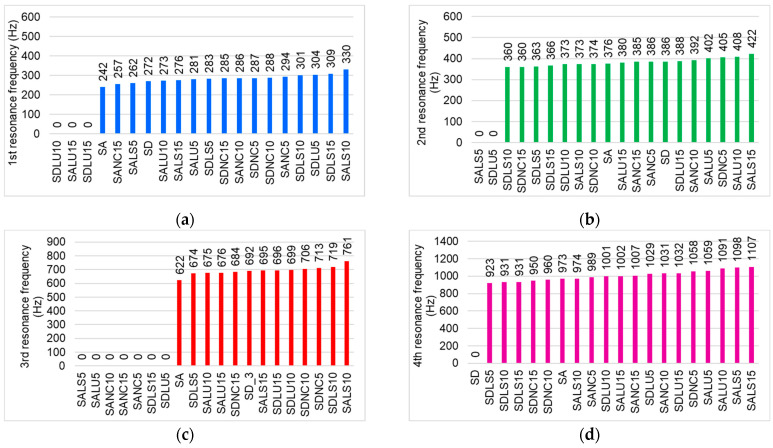

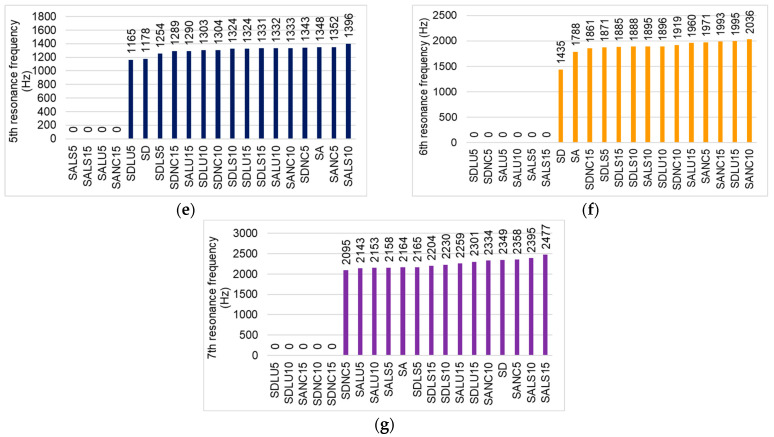
Values of resonance frequencies of studied plates: (**a**) first resonance frequency from 240 to 350 Hz; (**b**) second resonance frequency from 350 to 450 Hz; (**c**) third resonance frequency from 610 to 760 Hz; (**d**) fourth resonance frequency from 920 to 1120 Hz; (**e**) fifth resonance frequency from 1150 to 1400 Hz; (**f**) sixth resonance frequency from 1400 to 2058 Hz; (**g**) seventh resonance frequency from 2090 to 2600 Hz.

The natural frequencies of the tested plates depend on the anatomical structure associated with their quality grade, as well as the type and thickness of the varnish. Figure 6 selectively presents the frequency spectra of both unvarnished plates and plates varnished with five layers from each varnish category and anatomical class. The fundamental frequency is defined as the first frequency in the spectrum. For the unvarnished plates (Figure 6a,b), the fundamental frequency also corresponds to the dominant frequency. In contrast, for the varnished plates, the dominant frequency often corresponds to a higher-order harmonic within the frequency spectrum. All peaks representing resonance frequencies—associated with different vibration modes—were extracted for each tested plate. The values of these frequencies, categorized by interval, are displayed in the graphs shown in Figure 7.

Figure 7 illustrates the main resonance frequency ranges in relation to the characteristic vibrational modes of violins (modes A_0_, CBR, C4, B⁺, B^−^) [15,16,19,20]. The analyzed plates are arranged in ascending order according to the resonance frequency value for each corresponding mode. In Figure 7a, the first resonance frequencies of the tested boards are shown. The highest values for the first frequency are observed in samples coated with spirit and nitrocellulose varnishes. These frequencies correspond to the A_0_ mode of violin bodies [14,15,17,18,19]. Notably, the boards varnished with 10 and 15 layers of oil-based varnish do not exhibit resonance within the 240–330 Hz range. In the subsequent range, 360–422 Hz—corresponding to the CBR (Center Bout Rotation) mode—it is observed that some boards, despite differences in varnish type, layer thickness, or wood quality, share identical resonance frequencies. For example, boards varnished with 15 layers of nitrocellulose varnish and 10 layers of spirit varnish, both made from class D spruce, exhibit a frequency of 360 Hz. Similarly, boards coated with 10 layers of oil-based varnish (class D) and 10 layers of spirit varnish (class A) show the same resonance frequency of 376 Hz. Additionally, both the SANC5 and SD boards show a frequency of 386 Hz. This suggests that a specific combination of anatomical wood structure, varnish type, and coating thickness can produce identical resonance behavior.

The highest frequency in this second vibration mode is observed in sample SALS15 at 422 Hz (Figure 7b). The next resonance frequency range, 622–761 Hz, corresponds to the C4 mode (Figure 7c). Not all boards exhibit resonances within this range—for instance, class A boards varnished with 5 layers (regardless of varnish type), boards coated with 10 and 15 layers of nitrocellulose varnish, and class D boards with 10 layers of spirit varnish show no resonance here. The highest frequencies in this range are found in class D boards varnished with 10 layers of alcohol-based or nitrocellulose varnish. The fourth vibration mode, with resonance frequencies between 923 and 1107 Hz (Figure 7d), is present across all boards and is dominated by the varnished plates. A clear pattern emerges: for this mode, resonance frequencies tend to polarize based on the wood quality class and varnish type. Class D boards varnished with nitrocellulose exhibit the lowest frequencies, followed by those coated with spirit varnish. In contrast, boards varnished with oil-based varnish tend to show dominant frequencies above 1029 Hz. These findings align with conclusions from [30], which observed that alcohol-based varnish contributes more to damping than to stiffness, and that coating both surfaces of spruce significantly reduces its acoustic (radiation) properties. For the higher-order modes, it is observed that the ability to dampen high frequencies depends strongly on the type of finish applied to the plates (Figure 7e–g).

**Figure 8:**
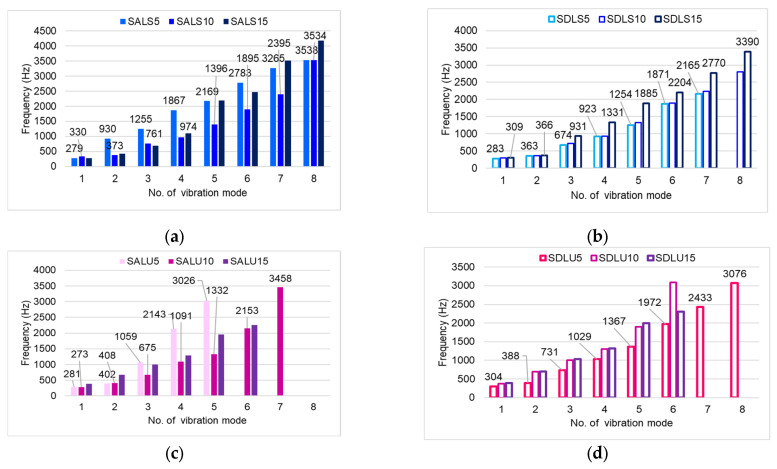

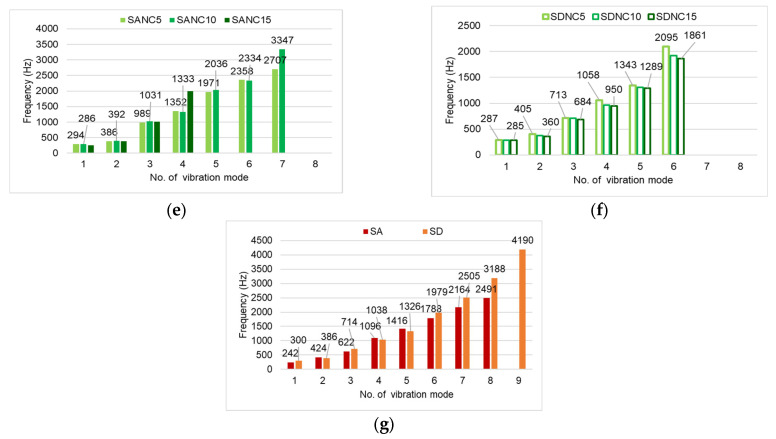
Distribution in frequency of the first eight peaks in ascending order of studied plates: (**a**) spruce plates grade A, spirit varnish; (**b**) spruce plates grade D, spirit varnish; (**c**) spruce plates grade A, oil-based varnish; (**d**) spruce plates grade D, oil-based varnish; (**e**) spruce plates grade A, nitrocellulose varnish; (**f**) spruce plates grade D, nitrocellulose varnish; (**g**) unvarnished spruce plates grade A and D.

The influence of lacquer film thickness on the vibration modes and resonance frequency spectrum of the tested plates is illustrated in Figure 8. In terms of the number of harmonics, boards varnished with spirit varnish exhibit the richest frequency spectra, regardless of the varnish thickness or the quality class of the spruce wood (Figure 8a,b). For oil-based varnish, the most favorable combinations are class A wood with a film thickness of 10 layers, and class D wood with a film of 5 layers (Figure 8c,d). In contrast, a thick nitrocellulose varnish layer results in a reduction in the frequency spectrum in class A spruce boards. For class D wood, a general decrease in resonance frequencies is observed as the varnish film thickness increases (Figure 8e,f). Compared to unvarnished wood (Figure 8g), which exhibits the richest frequency spectrum overall, the application of varnish consistently leads to an increase in the fundamental frequency value.

### 3.2. Damping of Varnished Plates

**Figure 9:**
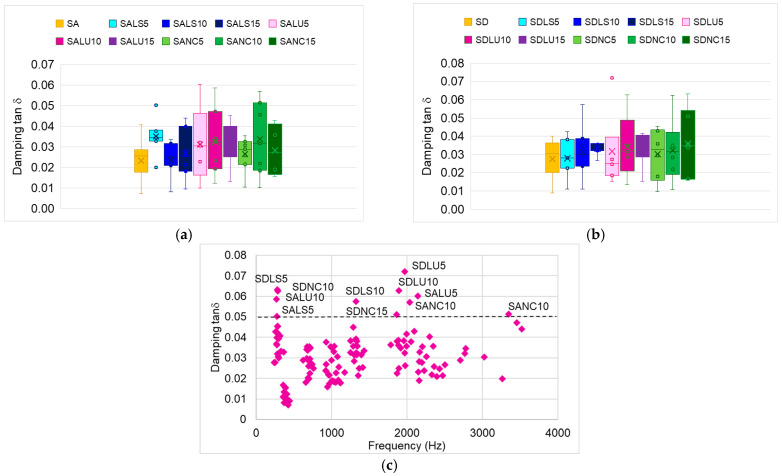
Variability of damping: (**a**) for plates made from spruce wood grade A; (**b**) for plates made from spruce wood grade D; (**c**) with frequency.

Analysis of the frequency spectrum reveals that the evaluated parameters influence the acoustic spectrum in different ways, highlighting the need to examine the vibration damping coefficient. Since previous studies have shown that surface finishing increases the mass of the sample, the radial stiffness, and internal friction, this study analyzed spruce wood boards with various finishes and thicknesses. Figure 9a,b illustrates the variability of the damping coefficient, expressed as tan δ. It is evident that varnished boards exhibit higher damping values compared to unvarnished boards. The greatest variability in damping values is observed in class A boards varnished with oil-based varnish (10 and 15 layers), as well as in class D boards finished with 5 and 10 layers of oil-based varnish and those coated with nitrocellulose varnish.

Regarding vibration damping across frequency ranges, Figure 9c shows that for frequencies below 1000 Hz, damping exceeds 0.05 for the SDLS5, SALS5, SALU10, and SDNC10 boards. In the 1–2 kHz range, damping is greater than 0.05 for the SDLS10, SDNC15, SDLU10, and SDLU5 boards. For high frequencies above 2 kHz, elevated damping values are observed in the SALU5 and SANC10 boards. In contrast, the remaining board types show damping values below 0.05 across the entire frequency range analyzed.

A low tan δ is one of the key conditions for ensuring the acoustic quality of resonance plates. The minimum and maximum values of the damping coefficient, along with the frequencies at which these values were recorded, are summarized in Table 3. The lowest damping values are found in uncoated plates, while the highest maximum damping values occur in plates varnished with oil-based varnish (SDLU5, SDLU10) and nitrocellulose varnish (SDNC10, SDNC15).

### 3.3. Quality Factor

**Figure 10:**
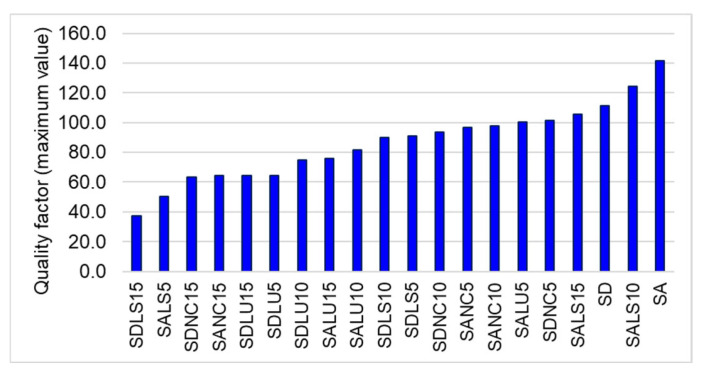
Variation in quality factors of tested plates.

The quality factor (Q) was calculated for the dominant frequency of each board, and the results are presented in ascending order in Figure 10. The highest Q values—exceeding 100—were recorded for the unvarnished SA and SD boards, as well as for SALS10, SALS15, and SDNC5. High-quality factor values, ranging between 80 and 100, were also observed in boards such as SALU10, SDLS10, SDLS5, SDNC10, SANC5, and SANC10. The remaining boards showed Q values below 80, with the lowest values corresponding to those varnished with spirit or nitrocellulose varnish in 10 and 15 layers.

**Figure 11:**
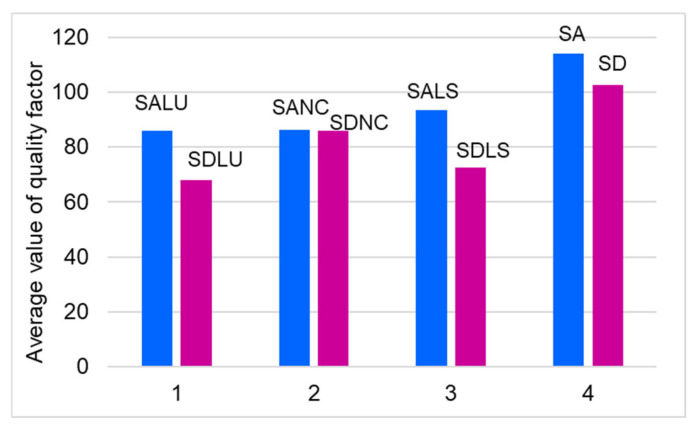
The influence of varnish on quality factor of spruce wooden plates.

Quantitatively, the quality factor of class A resonance spruce was found to be approximately 10% higher than that of class D wood (Figure 11). The application of varnish generally reduces the quality factor, with the extent of reduction depending on the type of solvent used. The greatest decrease in Q was observed with oil-based varnish—approximately 33% for class D and 25% for class A. This was followed by nitrocellulose varnish (24% for class A and 16% for class D), and alcohol-based varnish (18% for class A and 29% for class D). These results offer practical guidance for violin makers, who can select the optimal combination of varnish type and wood quality to minimize adverse effects on the quality factor. Based on the findings, the best combinations are class A wood with oil-based or spirit varnish, and class D wood with nitrocellulose varnish.

According to studies [14,15,18,19] on violins in the white (unvarnished) state and those varnished with spirit- and oil-based varnishes, varnishing does not alter the mode shapes but does influence the modal frequencies—particularly the B(1^−^) and B(1⁺) modes. Violin plates varnished with spirit varnish exhibit higher modal frequencies compared to those coated with oil-based varnish.

### 3.4. Sound Absorption Coefficient

**Figure 12:**
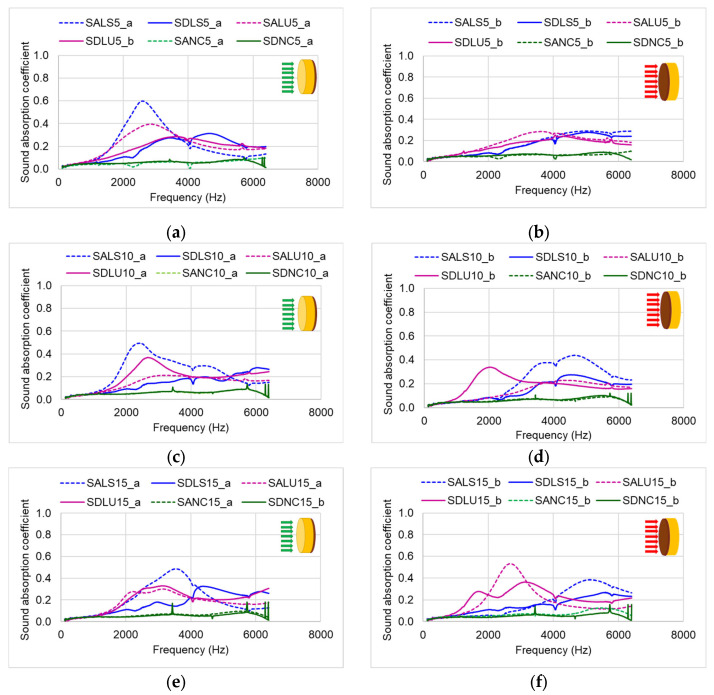
Average values of the acoustic absorption coefficient depending on the type of varnish applied to the wood samples: (**a**) 5-layer thickness, with the unvarnished side exposed; (**b**) 5-layer thickness, with the varnished side exposed; (**c**) 10-layer thickness, with the unvarnished side exposed; (**d**) 10-layer thickness, with the varnished side exposed; (**e**) 15-layer thickness, with the unvarnished side exposed; (**f**) 15-layer thickness, with the varnished side exposed.

**Figure 13:**
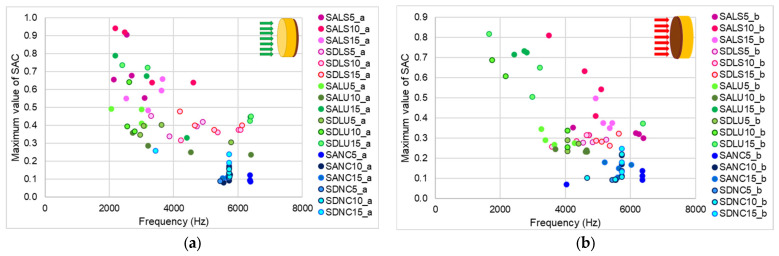
Maximum values of the sound absorption coefficient in relation to frequency: (**a**) unvarnished side in case of studied samples; (**b**) varnished side in case of studied samples.

Based on the experimental data, the sound absorption coefficient (SAC) was determined, as shown in Figure 12. Regardless of which side was tested, the SAC values remained below 0.6. However, compared to unvarnished wood, the varnished samples exhibited SAC values approximately 25–30% lower than those of the unvarnished side. When comparing the different sample types, the lowest SAC values were observed in samples varnished with nitrocellulose varnish, regardless of which side was exposed to the incident acoustic energy. For samples varnished with oil-based and spirit varnishes, the unvarnished side consistently showed higher SAC values than the varnished side. Additionally, the maximum acoustic absorption for the varnished surfaces occurred at higher frequencies, around 4 kHz. The acoustic behavior also varies depending on the anatomical structure of the wood. Although the proportion of latewood and earlywood is approximately the same in both quality classes, class D wood—characterized by annual ring widths approximately three times larger—exhibits different acoustic absorption capacities. These differences, however, are reduced by the application of the varnish film (Figure 13). The variations in absorption and reflection observed between the two surfaces—due to differences in structure, roughness, and porosity—are intentionally utilized in the construction of musical instruments with a resonant body. These surface-specific acoustic properties contribute to the instrument’s overall tonal quality.

### 3.5. Sound Reflection Coefficient

**Figure 14:**
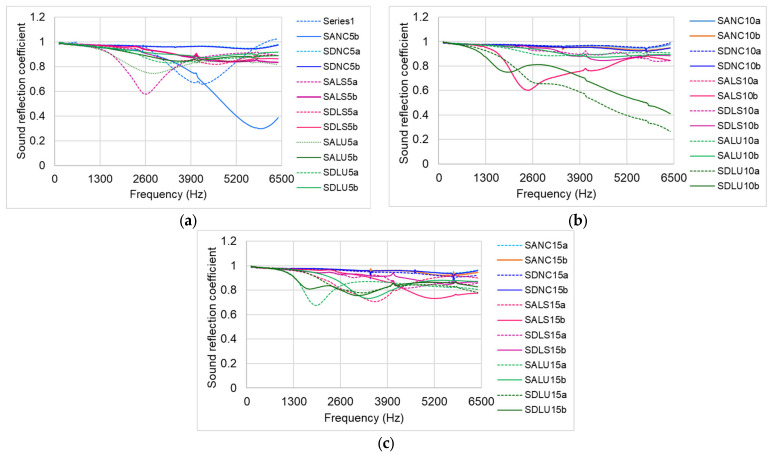
Sound reflection coefficient: (**a**) 5-layer thickness; (**b**) 10-layer thickness; (**c**) 15-layer thickness.

Since the impedance tube is considered an acoustically closed system, any portion of the incident wave that is not reflected by the material must be absorbed. For materials used in violin construction, it is therefore essential to understand both the acoustic absorption coefficient and the reflection coefficient. As shown in Figure 14, the reflection coefficients of samples varnished with different types of varnish range from 0.6 to 1. An increase in varnish film thickness corresponds to an increase in sound reflection, indicating that thicker coatings enhance the material’s reflective properties.

Thus, sound reflection depends on the thickness and density of the bi-material system formed by the wood and the finish. The high sound reflection observed in samples varnished with nitrocellulose and alcohol-based varnishes can be attributed to the properties of the solvents used. In contrast, the lowest reflection coefficient values are recorded for samples varnished with oil-based varnish. Because the drying process at the wood–finish interface takes longer for oil-based varnishes, the acoustic properties may continue to change over time, warranting further analysis of these parameters in the long term.

**Figure 15:**
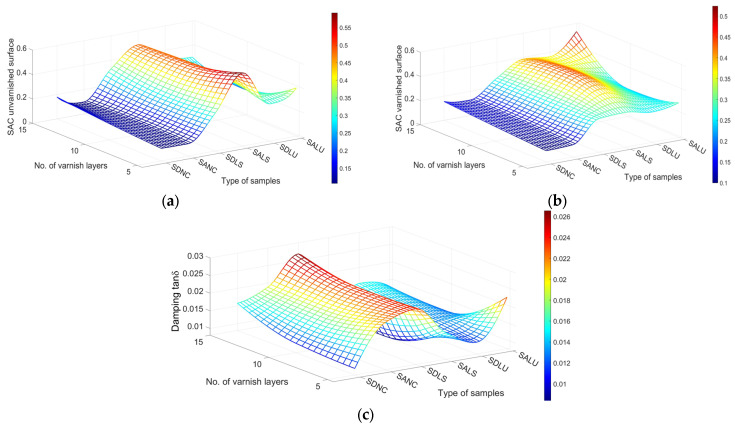
Variation in acoustic parameters: (**a**) sound absorption coefficient for unvarnished surface of samples; (**b**) sound absorption coefficient for varnished surface of samples; (**c**) damping of varnished samples.

Figure 15 illustrates the relationship between varnish type, coating thickness, and spruce wood grade for three key acoustic parameters: the absorption coefficient of the unvarnished surface (Figure 15a), the absorption coefficient of the varnished surface (Figure 15b), and the damping of the dominant frequency (Figure 15c). Across all parameters, nitrocellulose varnish consistently yields the lowest values, while spirit varnish shows the opposite trend, producing the highest values.

## 4. Conclusions

In this study, the acoustic properties of unvarnished and varnished spruce resonance wood—coated with three different types of varnishes commonly used in musical instrument manufacturing—were investigated from a new perspective. Modal analysis was employed to determine resonance frequencies, quality factors, and damping, while the impedance tube method was used to measure sound absorption and reflection coefficients. The key findings are as follows:

- The varnish film alters the frequency spectrum, both in terms of the number and values of resonant frequencies, due to increased stiffness of the plates. The fundamental frequency is higher than that of unvarnished wood.
- Unvarnished spruce wood exhibits the highest quality factor (Q). The quality factor is influenced by the anatomical quality of the spruce wood in correlation with the type of varnish applied.
- The highest damping coefficient values were observed in class D spruce boards varnished with oil-based and nitrocellulose varnishes. The highest acoustic absorption coefficients were found in samples varnished with 15 layers of oil-based varnish at 2672 Hz, and in those coated with 10 layers of spirit varnish at 4650 Hz.
- The richest frequency spectrum was recorded in boards coated with spirit varnish, though all samples exhibited frequencies within the 270–3500 Hz range.
- The sound absorption of unvarnished surfaces is significantly affected by the presence of varnish on the opposite surface.
- The highest reflection coefficient values were recorded in samples varnished with nitrocellulose varnish.

However, due to the slow and varying drying processes of varnish within the wood, these acoustic parameters may change over time. As such, it is recommended that measures be repeated after a minimum of 6 months, as suggested by previous research in the field. Future research will focus on two main directions: rheological analysis of acoustic parameters over time, and the acoustic evaluation of violins varnished with these different varnish types.

## Figures and Tables

**Table 1:** The anatomical features of the spruce wood sample from two different quality grades [31].

Physical Features	Grade/Average Value/STDV			
	A	STDV	D	STDV
Annual rings width (mm)	0.71	0.005	2.28	0.005
Early wood width (mm)	0.54	0.011	1.74	0.029
Late wood width (mm)	0.18	0.013	0.54	0.026
Early wood proportion (%)	74.97	1.519	76.36	1.138
Late wood proportion (%)	25.03	1.519	23.64	1.136
Density *ρ* (kg/m^3^)	409.5	28.50	420.00	26.00

**Table 2:** The physical features of the cylindrical varnished samples (Legend: SALU5—spruce wood grade A, varnished with oil-based varnish, 5 layers; SALS5—spruce wood grade A, varnished with spirit varnish, 5 layers; SANC5—spruce wood grade A, varnished with nitrocellulose varnish, 5 layers; SALU10—spruce wood grade A, varnished with oil-based varnish, 10 layers; SALS10—spruce wood grade A, varnished with spirit varnish, 10 layers; SANC10—spruce wood grade A, varnished with nitrocellulose varnish, 10 layers; SALU15—spruce wood grade A, varnished with oil-based varnish, 15 layers; SALS15—spruce wood grade A, varnished with spirit varnish, 15 layers; SANC15—spruce wood grade A, varnished with nitrocellulose varnish, 15 layers).

Type of Varnished Samples	No. of Samples	Thickness of Varnish Layer (μm)	Mass (g)
SALU5	4	40 ± 10	1.146
SALS5			1.167
SANC5			1.348
SALU10	4	80 ± 10	1.322
SALS10			1.188
SANC10			1.449
SALU15	4	130 ± 10	1.376
SALS15			1.327
SANC15			1.517
SDLU5	4	35 ± 10	1.186
SDLS5			1.125
SDNC5			1.144
SDLU10	4	70 ± 10	1.258
SDLS10			1.168
SDNC10			1.215
SDLU15	4	120 ± 10	1.337
SDLS15			1.189
SDNC15			1.443

**Table 3:** Summary of mean, standard deviations minimum and maximum values of damping coefficient.

Samples	Average Value of Damping & STDV	Minimum Value of Damping tan δ	Frequency (Hz) for Minim tan δ	Maximum Value of Damping tan δ	Frequency (Hz) for Maxim tan δ
SA	0.023 (0.0099)	0.007	425	0.041	319
SALS5	0.035 (0.0090)	0.020	3265	0.050	279
SALS10	0.024 (0.0083)	0.008	373	0.033	330
SALS15	0.027 (0.0120)	0.009	422	0.040	276
SALU5	0.031 (0.0180)	0.010	402	0.060	2143
SALU10	0.032 (0.0160)	0.012	408	0.059	273
SALU15	0.032 (0.0110)	0.013	380	0.045	1290
SANC5	0.026 (0.0080)	0.010	386	0.035	989
SANC10	0.034 (0.0180)	0.010	392	0.057	2036
SANC15	0.028 (0.0130)	0.016	385	0.043	257
SD	0.028 (0.0100)	0.009	446	0.040	300
SDLS5	0.028 (0.0100)	0.011	363	0.042	283
SDLS10	0.031 (0.0150)	0.011	360	0.057	1324
SDLS15	0.033 (0.0033)	0.027	931	0.036	1331
SDLU5	0.032 (0.0190)	0.015	388	0.072	1972
SDLU10	0.034 (0.0180)	0.013	373	0.063	1896
SDLU15	0.034 (0.0097)	0.015	388	0.042	1995
SDNC5	0.030 (0.0140)	0.010	405	0.045	287
SDNC10	0.032 (0.0170)	0.011	374	0.063	288
SDNC15	0.036 (0.0190)	0.016	950	0.063	285

## Data Availability

The original contributions presented in this study are included in the article. Further inquiries can be directed to the corresponding author.
